# Meta-Analysis of Early Nutrition: The Benefits of Enteral Feeding Compared to a Nil Per Os Diet Not Only in Severe, but Also in Mild and Moderate Acute Pancreatitis

**DOI:** 10.3390/ijms17101691

**Published:** 2016-10-20

**Authors:** Katalin Márta, Nelli Farkas, Imre Szabó, Anita Illés, Áron Vincze, Gabriella Pár, Patrícia Sarlós, Judit Bajor, Ákos Szűcs, József Czimmer, Dóra Mosztbacher, Andrea Párniczky, Kata Szemes, Dániel Pécsi, Péter Hegyi

**Affiliations:** 1 Institute for Translational Medicine, University of Pécs, Pécs H-7624, Hungary; katalin.martak@gmail.com (K.M.); nelli.farkas@aok.pte.hu (N.F.); sarlos.patricia@pte.hu (P.S.); bajor.judit8@gmail.com (J.B.); szucs.akos@gmail.com (A.S.); dora.mosztbacher@gmail.com (D.M.); andrea.parniczky@gmail.com (A.P.); szemesk@gmail.com (K.S.); daniel.pecsi1991@gmail.com (D.P.); 2 Institute of Bioanalysis, University of Pécs, Pécs H-7624, Hungary; 3 Department of Gastroenterology, First Department of Medicine, University of Pécs, Pécs H-7624, Hungary; szaboimi@yahoo.com (I.S.); anitai@freemail.hu (A.I.); vincze.aron@pte.hu (A.V.); pargabriella@gmail.com (G.P.); czimmer.jozsef@pte.hu (J.C.); 4 First Department of Surgery, Semmelweis University, Budapest H-1085, Hungary; 5 First Department of Pediatrics, Semmelweis University, Budapest H-1083, Hungary; 6 Heim Pál Children’s Hospital, Budapest H-1089, Hungary; 7 Translational Gastroenterology Research Group, Hungarian Academy of Sciences, University of Szeged, Szeged H-6720, Hungary; 8 First Department of Medicine, University of Szeged, Szeged H-6720, Hungary

**Keywords:** enteral feeding, acute pancreatitis, early nutrition, energy, meta-analysis

## Abstract

The recently published guidelines for acute pancreatitis (AP) suggest that enteral nutrition (EN) should be the primary therapy in patients suffering from severe acute pancreatitis (SAP); however, none of the guidelines have recommendations on mild and moderate AP (MAP). A meta-analysis was performed using the preferred reporting items for systematic review and meta-analysis protocols (PRISMA-P). The following PICO (problem, intervention, comparison, outcome) was applied: P: nutrition in AP; I: enteral nutrition (EN); C: nil per os diet (NPO); and O: outcome. There were 717 articles found in Embase, 831 in PubMed, and 10 in the Cochrane database. Altogether, seven SAP and six MAP articles were suitable for analyses. In SAP, forest plots were used to illustrate three primary endpoints (mortality, multiorgan failure, and intervention). In MAP, 14 additional secondary endpoints were analyzed (such as CRP (C-reactive protein), WCC (white cell count), complications, etc.). After pooling the data, the Mann–Whitney *U* test was used to detect significant differences. Funnel plots were created for testing heterogeneity. All of the primary endpoints investigated showed that EN is beneficial vs. NPO in SAP. In MAP, all of the six articles found merit in EN. Analyses of the primary endpoints did not show significant differences between the groups; however, analyzing the 17 endpoints together showed a significant difference in favor of EN vs. NPO. EN is beneficial compared to a nil per os diet not only in severe, but also in mild and moderate AP.

## 1. Introduction

Acute pancreatitis (AP) is a severe inflammatory disease with high mortality [1]. Despite the extensive research in the field, no specific therapy is available to treat AP [2]. With regard to the pathomechanism of the disease, it is clear that mitochondrial injury and ATP depletion play key roles in the early phase of AP almost irrespectively of the etiology of the disease [3,4,5]. Bile acids, ethanol, and fatty acids were shown to be responsible for around 80% of the etiological factors initiating AP [6]. All of these factors were shown to induce a toxic calcium signal and severe mitochondrial damage in both acinar and ductal cells [3,7,8,9,10,11]. Importantly, direct administration of ATP (i.e., energy) into the cells restored their functions and prevented cell death [12,13]. Therefore, if we take a translational approach, it is more than likely that patient energy intake would be beneficial. Not surprisingly, enteral nutrition (EN) has almost been the only therapeutic change in recent decades to be highly beneficial and to be widely utilized in severe AP (SAP) [14]. However, in mild and moderate AP (MAP), the primary therapy is still the nil per os diet (NPO) [15]. Since the results in basic science have demonstrated the crucial role of energy breakdown in the early phase of AP, in this study we performed a systemic review of the literature followed by a meta-analysis to understand whether enteral feeding should be the primary therapy not only in severe AP, but in mild and moderate AP as well.

## 2. Results

### 2.1. Severe Acute Pancreatitis (SAP) Group

**Figure 1:**
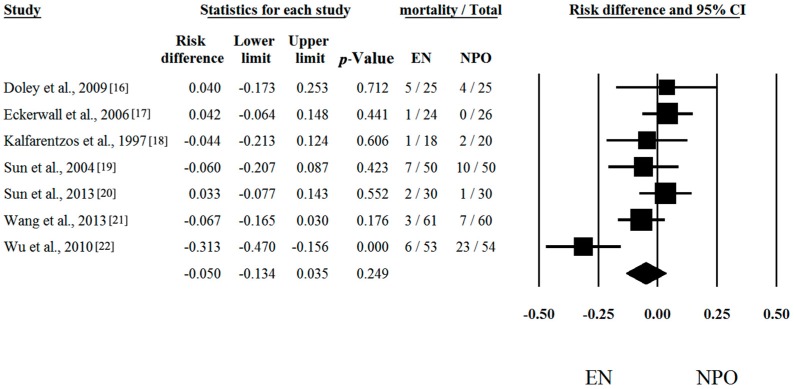
Forest plot of studies evaluating mortality data in severe acute pancreatitis (SAP). Risk differences and confidence interval (CI) were calculated to compare the enteral nutrition (EN) with the nil per os diet (NPO). Black squares and lines represent the results for individual studies, the diamond shows the pooled result of the meta-analysis.

**Figure 2:**
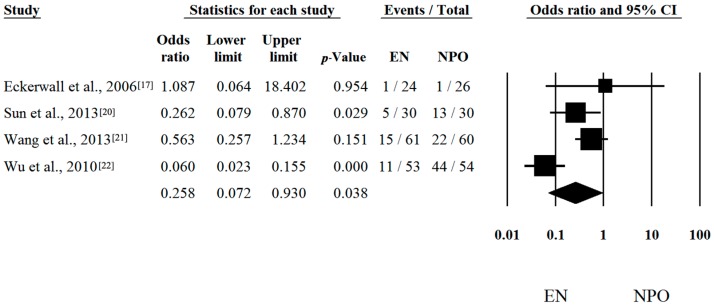
Forest plot of studies evaluating multiorgan failure (MOF) in severe acute pancreatitis (SAP). Odds ratio (OR) and confidence interval (CI) were calculated to compare the enteral nutrition (EN) with the nil per os diet (NPO). Black squares and lines represent the results for individual studies, the diamond shows the pooled result of the meta-analysis.

**Figure 3:**
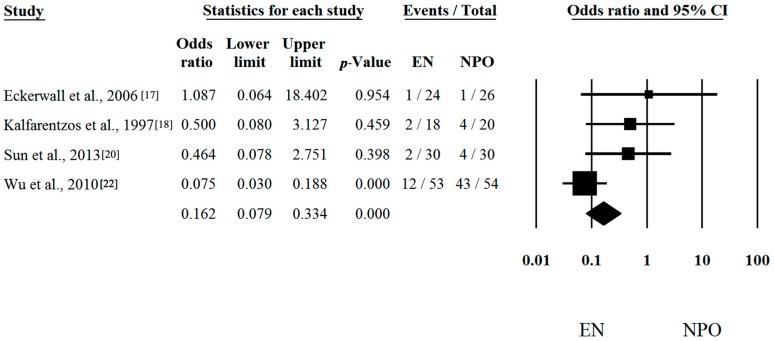
Forest plot of studies evaluating intervention in severe acute pancreatitis (SAP). Odds ratio (OR) and confidence interval (CI) were calculated to compare the enteral nutrition (EN) with the nil per os diet (NPO). Black squares and lines represent the results for individual studies, the diamond shows the pooled result of the meta-analysis.

Seven out of seven articles contained analyzable data on mortal [16,17,18,19,20,21,22] Risk differences and CI were calculated in each article to analyze the effects of EN compared to the NPO nutrition. The calculated average risk difference (RD) was −0.050 (lower limit (LI): −0.134; upper limit (UI): 0.035; *p*-value: 0.249) (Figure 1). Because of the considerable heterogeneity (Q = 16.488; DF: 6; *p* = 0.011; I^2^ = 63.61%) random-effect model was applied. Four out of seven articles contained analyzable data on multiorgan failure (MOF). With regard to MOF, the calculated odds ratio (OR) was 0.258 (LI: 0.072; UI: 0.930; *p*-value: 0.038; heterogeneity: Q = 13.833; DF: 3; *p* = 0.003; I^2^ = 78.31%) in favor of EN (Figure 2). With regard to interventions, a fixed-effect model was used. The calculated average odds ratio (OR) was 0.162 (LI: 0.079; UI: 0.334; *p*-value: <0.001; Q = 7.221; DF: 3; *p* = 0.065; I^2^ = 58.45%) also in favor of EN (Figure 3). Because of the moderate heterogeneity, the random-effect model was applied as well (OR was 0.274 (LI: 0.073; UI: 1.025; *p* = 0.054)). These data clearly suggest that EN is beneficial and should be the primary therapy in SAP.

### 2.2. Mild and Moderate Acute Pancreatitis (MAP) Group

**Figure 4:**
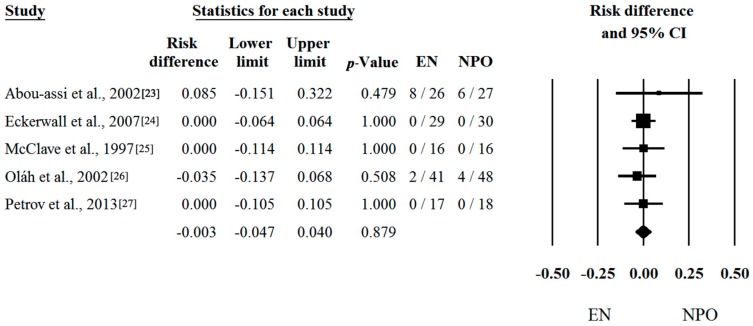
Forest plot of studies evaluating mortality data in mild and moderate acute pancreatitis (MAP). Risk differences and confidence interval (CI) were calculated to compare the enteral nutrition (EN) with the nil per os diet (NPO). Black squares and lines represent the results for individual studies, the diamond shows the pooled result of the meta-analysis.

**Figure 5:**
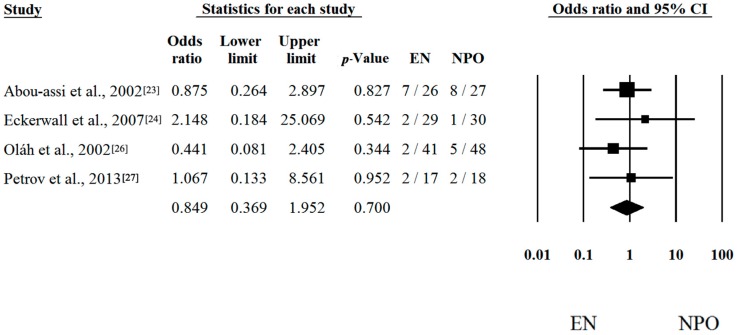
Forest plot of studies evaluating multiorgan failure (MOF) in mild and moderate acute pancreatitis (MAP). Odds ratio (OR) and confidence interval (CI) were calculated to compare the enteral nutrition (EN) with the nil per os diet (NPO). Black squares and lines represent the results for individual studies, the diamond shows the pooled result of the meta-analysis.

Unfortunately, there is much less research activity in patients suffering from MAP than from SAP. Moreover, the frequency of death and MOF are also much less common in the MAP group vs. the SAP group. Not surprisingly, analyses of low amounts of data in which the mortality and MOF are close to zero could not reveal any significant difference between the two groups. With regard to mortality, five out of six articles contained proper data [23,24,25,26,27]. Risk differences and CI were calculated in the articles. The calculated average risk difference (RD) was −0.003 (LI: −0.047; UI: 0.040; *p*-value: 0.879) (Figure 4). As predicted, we also saw no significant difference in the frequency of MOF, where we only had four items. Forest plots of OR and CI were calculated. The odds ratio (OR) was 0.849 (LI: 0.369; UI: 1.952; *p*-value: 0.700) (Figure 5). Because of the Q and I^2^ tests showed negligible heterogeneity (Q = 0.916; DF: 4; *p* = 0.922; I^2^ = 0.00% for Figure 4 and Q = 1.169; DF: 3; *p* = 0.760; I^2^ = 0.00% for Figure 5), the fixed-effect model was applied.

**Figure 6:**
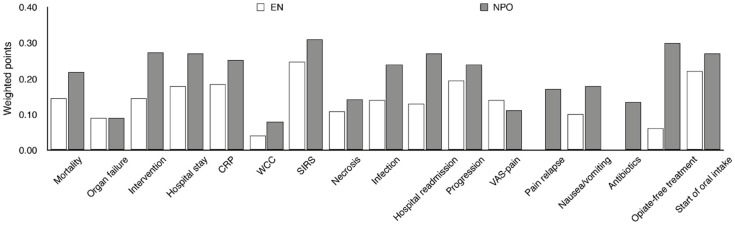
Summary of the uniform data-point system in MAP. EN vs. NPO. Due to the low amount of data, 3 primary endpoints and 14 secondary endpoints were collected for MAP. The uniform data point system was then developed (Table 1). Results were weighted based on the number of patients in the articles. CRP, C-reactive protein; WCC, white cell count; SIRS, systemic inflammatory response syndrome; VAS, visual analogue scale.

**Figure 7:**
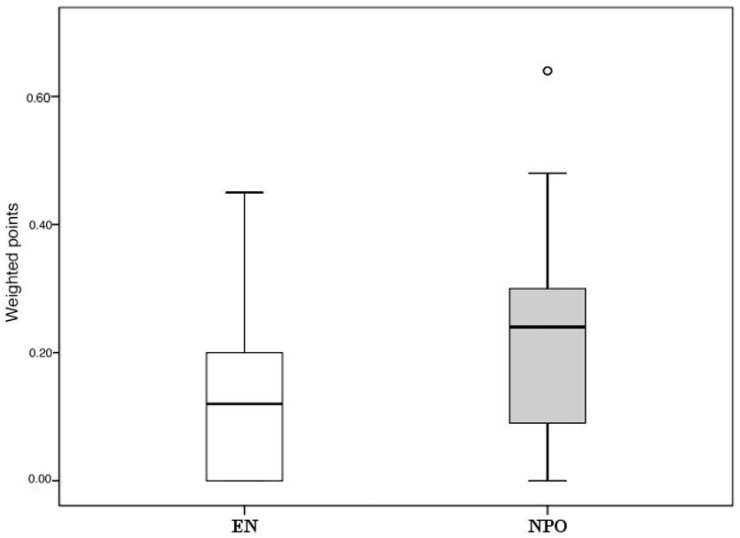
Summary of the uniform data point system in MAP. EN vs. NPO. The Mann–Whitney U test was used to detect significant differences between the pooled weighted scores (see Figure 6). o = *p* < 0.05 vs. EN.

However, the five articles contained several other secondary parameters (see Methods). Unfortunately, each study group concentrated on different parameters, resulting in the fact that almost none of the parameters had a complete data set (Figure S1). Figure 6 demonstrates the differences between EN and NPO. Due to the low n number, statistical analyses could not be calculated separately. Importantly, pooling the data from the 17 parameters (3 primary and 14 secondary endpoints) showed a significant difference in favor of EN (Figure 7). The significant difference was also observed when different powers (when primary endpoints were double weighted) of the endpoints were applied. The supplementary data sheet contains all the data used for the statistical analyses.

## 3. Discussion

There are different therapeutic approaches available with regard to nutrition in acute pancreatitis. The recently published IAP/APA (International Association of Pancreatology/American Pancreatic Association) guidelines recommend that enteral tube feeding be the primary therapy in patients with predicted severe and severe acute pancreatitis who require nutritional support (recommendation G. Nutritional support 21-GRADE 1B, strong agreement), whereas point K22 in the Japanese guidelines states that enteral nutrition can reduce the incidence of complications in the early phase of SAP and can contribute to an increased rate of survival [2,28]. However, neither of the guidelines provides recommendations on MAP. The reason is understandable. (1) Strong endpoints are missing. The mortality rate is less than 1% in mild AP and 10% in moderate AP, whereas almost no MOF can be detected; (2) since there is a better outcome of the milder disease, researchers have had much less interest in MAP than SAP.

Here, we wanted to systematically review the current literature to understand the beneficial effects of early enteral nutrition vs. the nil per os diet both in SAP and MAP. Interestingly, there were not many articles in which analyzable data could be found on the two treatments of AP. However, in SAP, the amount of data was sufficient to prove the beneficial effects of enteral feeding. Early enteral feeding was clearly beneficial for MOF and intervention and showed beneficial tendency for mortality. Nevertheless, as predicted, MAP data analyses revealed no significant difference between enteral nutrition and a nil per os diet. However, analyses of the secondary endpoints in the articles demonstrated that enteral feeding could be beneficial compared to a nil per os diet in mild and moderate AP as well.

The six MAP studies applied different methods for enteral feeding. Eckerwall et al. [24] employed immediate oral feeding, Abou-Assi et al. [23], Oláh et al. [26], and McClave et al. [25] administered nasojejunal feeding, and Petrov et al. [27] and Ma et al. [29] used nasogastric feeding. Immediate oral feeding (EN) significantly cut the length of hospital stay without any adverse events [24]. Nasogastric feeding starting within 24 h of hospital admission was not only well tolerated, but also reduced the intensity and duration of abdominal pain, decreased the necessity of opiates, and almost totally eliminated the risk of oral food intolerance [27]. Moreover, patients in the nasogastric feeding group had significantly improved appetite vs. the NPO group [29]. Nasojejunal feeding lowers the stress response to AP [25] associated with a lower complication rate [26] and cuts the length of hospital stay. Importantly, the fact that all of the studies found merit in early enteral feeding in MAP suggests that it is not the way of feeding that is important, but the feeding itself, i.e., energy.

## 4. Materials and Methods

### 4.1. Article Search

A meta-analysis was performed using the preferred reporting items for systematic review and meta-analysis protocols (PRISMA-P) [30]. An article search was performed in the PubMed, EMBASE, and Cochrane databases in February 2016. The PICO process was used to frame and answer our clinical questions.

#### 4.1.1. PICO (Problem, Intervention, Comparison, Outcome)

PICO was broken down as follows: P: nutrition in AP; I: enteral nutrition; C: nil per os diet; and O: outcome. We split our data into two groups: SAP and MAP. In SAP, only three primary endpoints were checked (mortality, multiorgan failure, and intervention), whereas in MAP, due to the low amount of data, 14 secondary endpoints were collected besides the primary endpoints: length of hospital stay (LOH), inflammatory parameters (C-reactive protein (CRP), white cell count (WCC), and presence of SIRS (systemic inflammatory response syndrome)), complications (necrosis, infection, hospital readmission, and progression of severity), intervention, necessity of antibiotic, pain relapse, visual analogue scale (VAS)-pain, opiate-free treatment, start of oral intake, and clinical symptoms (nausea and vomiting).

#### 4.1.2. Search

**Figure 8:**
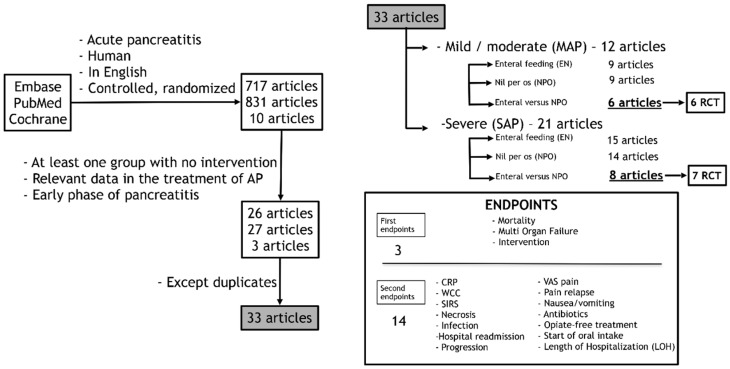
Organogram of article search in PubMed, EMBASE, and Cochrane databases. RCT, randomized and controlled trial; CRP, C-reactive protein; WCC, white cell count; SIRS, systemic inflammatory response syndrome; VAS, visual analogue scale.

A search was made using the following terms: in PubMed: (acute (All Fields) and “pancreatitis” (MeSH Terms) or “pancreatitis” (All Fields)) and (“clinical trial” (Publication Type) or “clinical trials as topic” (MeSH Terms) or “clinical trials” (All Fields)) and (“loattrfull text” (sb) and “humans” (MeSH Terms) and English (lang)) in EMBASE: “acute pancreatitis” and (humans)/lim and (English)/lim and (abstracts)/lim and ((controlled clinical trial)/lim or (randomized controlled trial)/lim) and in Cochrane: “acute pancreatitis”: ti,ab,kw and “human” and “English” in Trials (the search included various forms of the terms). “Acute pancreatitis” in Title, Abstract and Keywords and “human” and ”English” in Trials (the search included various forms of the terms). Altogether, 1634 articles (EMBASE: 717; PubMed: 831; Cochrane: 10) were found (Figure 8).

#### 4.1.3. Inclusions and Exclusions

A manual search was performed to find the relevant articles. Only articles in English and with relevant data in the early phase treatment of AP were included. Duplications were excluded. Thirty-three articles (21 articles containing patients suffering from SAP as well as 12 articles with MAP patients) were selected. They contained two nonrandomized and 31 randomized controlled clinical trials (Table 2) [16,17,18,19,20,21,22,23,24,25,26,27,29,31,32,33,34,35,36,37,38,39,40,41,42,43,44,45,46,47,48,49,50]. Finally, statistical analyses were performed on data from articles where both EN and NPO groups were presented, the trial was randomized, and the relevant data were available. Altogether, seven SAP and six MAP articles met these criteria.

#### 4.1.4. Statistical Analyses

In SAP, forest plots were used to illustrate the mortality, multiorgan failure and intervention. In the case of mortality and multiorgan failure, the pooled estimates were calculated with a random-effects model; in the case of intervention, a fixed-effects model was applied as described earlier [51]. Analyses were performed with the Comprehensive Meta-Analysis Software (Biostat, Inc., Englewood, NJ, USA). In the case of binary variables, the differences between EN and NPO were expressed as risk differences or odds ratios with a 95% confidence interval (CI). Heterogeneity was tested between trials with two methods. First, we employed the Q homogeneity test statistic, which exceeds the upper-tail critical value of chi-square on n − 1 degrees of freedom (DF), with a *p*-value of less than 0.050 considered suggestive of significant heterogeneity. Second, we used the inconsistency (I^2^) index. I^2^ is the proportion of total variation contributed by between-study variability. An I^2^ value of more than 0.5 suggests a considerable heterogeneity. Heterogeneity was verified using a funnel plot to reduce publication bias. Whenever considerable heterogeneity was observed, random- or fixed-effects models were applied.

In MAP, only two (mortality and multiorgan failure) of the three primary endpoints could be analyzed. With regard to the second endpoints, no forest plot analyses could be calculated due to insufficient data. A uniform point system was developed to make the data analyzable (Table 1). Results were also weighted based on the number of patients in the articles. The Mann–Whitney *U* test was used to detect significant differences between the pooled weighted scores. SPSS Statistical Software (version 20, IBM Corporation, Armonk, NY, USA) facilitated this analysis. A *p*-value less than 0.05 was considered as statistically significant, whereas a *p*-value between 0.1 and 0.05 was seen as a trend.

## 5. Conclusions

Unfortunately, there are several limitations of this study, therefore, the results of this meta-analysis should be interpreted with caution. The biggest limitation is the small number of studies included (especially in MAP) which caused higher heterogeneity. The low amount of extracted data from the articles caused further difficulties. In MAP, a uniform point system had to be developed to make the data analyzable. Since these limitations attenuate the strength of this meta-analysis, more high-quality randomized controlled clinical trials (RCTs) are still needed to propound more evidence on treatment decisions in MAP.

In conclusion, enteral feeding is beneficial compared to a nil per os diet not only in severe, but also in mild and moderate AP. Additional studies should be performed to understand whether energy supply or enteral passage is more important.

## Figures and Tables

**Table 1:** Uniform point system. CRP, C-reactive protein; WCC, white cell count; SIRS, systemic inflammatory response syndrome; LOH, length of hospitalization; VAS, visual analogue scale.

**Points**	**Mortality (%)**	**Organ Failure (%)**	**Intervention (%)**	**CRP (mg/L)**	**WCC (109/L)**	**SIRS (%)**
0	0–0.9	0–0.09	0–0.09	0–19.9	4000–9999.9	0–0.09
1	1–2.9	0.1–0.19	0.1–0.19	20–39.9	10,000–11,999	0.1–0.14
2	3–4.9	0.2–0.29	0.2–0.29	40–59.9	12,000–13,999	0.15–0.19
3	5–6.9	0.3–	0.3–0.39	60–79.9	14,000–15,999	0.2–0.24
4	7–8.9		0.4–0.49	80–99.9	16,000–17,999	0.25–0.29
5	9–		0.5–	100–	18,000–	0.3–
**Points**	**LOH (Days)**	**Necrosis (%)**	**Infection (%)**	**Hospital Readmission (%)**	**Progression of Severity (%)**	**Pain Relapse (%)**
0	0–4.9	0–0.09	0–0.09	0–0.04	0–0.04	0–0.09
1	5–9.9	0.1–0.19	0.1–0.19	0.05–0.06	0.05–0.06	0.1–0.19
2	10–12.4	0.2–0.29	0.2–	0.07–0.08	0.07–0.08	0.2–0.29
3	12.5–14.9	0.3–	–	0.09–0.10	0.09–0.10	0.3–0.39
4	15–19.9	–	–	0.11–	0.11–	0.4–
5	20–	–	–	–	–	–
**Points**	**VAS-Pain**	**Nausea/Vomiting (%)**	**Antibiotics (%)**	**Opiate-Free Treatment (%)**	**Start of Oral Intake (%)**	
0	0–1	0–0.18	0–0.09	0–0.09	0–0.04	
1	2–4	0.2–0.39	0.1–0.19	0.1–0.19	0.05–0.09	
2	5–7	0.4–0.59	0.2–0.29	0.2–0.29	0.1–0.14	
3	8–9	0.6–0.79	0.3–0.39	0.3–0.39	0.15–0.19	
4	–	0.8–	0.4–	0.4–0.49	0.2–0.24	
5	–	–	–	0.5–	0.25–	

**Table 2:** Articles with data on the early phase of AP. SAP: severe acute pancreatitis; MAP: mild and moderate AP; EN: enteral nutrition; NPO: nil per os diet; RCT: randomized controlled clinical trial.

Article	MAP	SAP	EN	NPO	RCT
Doley et al. 2009 [16]	–	✔	✔	✔	✔
Eckerwall et al. 2006 [17]	–	✔	✔	✔	✔
Kalfarentzos et al. 1997 [18]	–	✔	✔	✔	✔
Sun et al. 2004 [19]	–	✔	✔	✔	✔
Sun et al. 2013 [20]	–	✔	✔	✔	✔
Wang et al. 2013 [21]	–	✔	✔	✔	✔
Wu et al. 2010 [22]	–	✔	✔	✔	✔
Abou-assi et al. 2002 [23]	✔	–	✔	✔	✔
Eckerwall et al. 2007 [24]	✔	–	✔	✔	✔
McClave et al. 1997 [25]	✔	–	✔	✔	✔
Oláh et al. 2002 [26]	✔	–	✔	✔	✔
Petrov et al. 2013 [27]	✔	–	✔	✔	✔
Ma et al. 2016 [29]	✔	–	✔	✔	✔
Li et al. 2013 [39]	✔	–	✔	–	✔
Ockenga et al. 2002 [41]	✔	–	–	✔	✔
Pandey et al. 2004 [42]	✔	–	✔	–	✔
Pongratz et al. 2013 [45]	✔	–	–	✔	✔
Sathiaraj et al. 2008 [46]	✔	–	✔	–	✔
Wu et al. 2011 [49]	✔	–	–	✔	✔
Andersson et al. 2006 [31]	–	✔	–	✔	–
Bakker OJ et al. 2014 [32]	–	✔	✔	–	✔
Besselink et al. 2008 [33]	–	✔	✔	–	✔
Eatock et al. 2005 [34]	–	✔	✔	–	✔
He et al. 2004 [35]	–	✔	–	✔	✔
Karakan et al. 2007 [36]	–	✔	✔	–	✔
Kumar et al. 2006 [37]	–	✔	✔	–	✔
Kyhala et al. 2012 [38]	–	✔	–	✔	✔
Modena et al. 2006 [40]	–	✔	✔	✔	–
Pearce et al. 2006 [43]	–	✔	✔	–	✔
Pettila et al. 2010 [44]	–	✔	–	✔	✔
Singh et al. 2012 [47]	–	✔	✔	–	✔
Vege et al. 2015 [48]	–	✔	–	✔	✔
Zhao et al. 2013 [50]	–	✔	–	✔	✔

## Supplementary Materials

Supplementary materials can be found at www.mdpi.com/1422-0067/17/10/1691/s1.
