# Supplementary material for: Meta-Analysis of Early Nutrition: The Benefits of Enteral Feeding Compared to a Nil Per Os Diet Not Only in Severe, but Also in Mild and Moderate Acute Pancreatitis
Source: Int J Mol Sci. 2016 Oct 20;17(10):1691. doi: 10.3390/ijms17101691 (PMC5085723; doi:10.3390/ijms17101691)
Supplement: Supplementary file 1 [file ijms-17-01691-s001.pdf]

Supplementary Materials: Meta-Analysis of Early Nutrition: The Benefits of Enteral Feeding Compared to a Nil Per Os Diet Not Only in Severe, but Also in Mild and Moderate Acute Pancreatitis

Katalin Márta, Nelli Farkas, Imre Szabó, Anita Illés, Áron Vincze, Gabriella Pár, Patrícia Sarlós, Judit Bajor, Ákos Szűcs, József Czimmer, Dóra Mosztbacher, Andrea Párniczky, Kata Szemes, Dániel Pécsi and Péter Hegyi

Table S1. Original data, points and weighted points in MAP. Data collected from the six MAP articles. Points were calculated based on the uniform point system (Table 2). Weighted points were calculated based on the points and number of patients. CRP, C-reactive protein; WCC, white cell count; SIRS, systemic inflammatory response syndrome; MOF, multi organ failure; VAS, visual analogue scale.

Study Name	Abou-assi et al. 2002 [23]		Eckerwall et al. 2007 [24]		McClave et al. 1997 [25]		Oláh et al. 2002 [26]		Petrov et al. 2013 [27]	
Type of nutrition	EN	NPO	EN	NPO	EN	NPO	EN	NPO	EN	NPO
Patients No (n)	26	27	29	30	16	16	41	48	17	18
multiplier	0.09	0.09	0.10	0.10	0.05	0.05	0.14	0.16	0.06	0.06
Mortality (n)	8	6	0	0	0	0	2	4	0	0
points	5	5	0	0	0	0	2	4	0	0
weighted points	0.45	0.45	0	0	0	0	0.28	0.64	0	0
Lenght of hospitalisation (days)	14.2	18.4	4	6	11.9	9.7	–	–	9	8.5
points	3	4	0	1	2	1	–	–	1	1
weighted points	0.27	0.36	0	0.10	0.10	0.05	–	–	0.06	0.06
CRP (mg/L)	–	–	61	81	98	106	–	–	44	66
points	–	–	3	4	4	5	–	–	2	3
weighted points	–	–	0.30	0.40	0.20	0.25	–	–	0.12	0.18
WCC (109/L)	–	–	6.6	7.7	–	–	–	–	10	12
points	–	–	0	0	–	–	–	–	1	2
weighted points	–	–	0	0	–	–	–	–	0.06	0.12
SIRS (n)	–	2	–	–	–	–	5	13	6	6
points	–	0	–	–	–	–	1	4	5	5
weighted points	–	0	–	–	–	–	0.14	0.64	0.3	0.3
Necrosis (n)	–	–	0	0	–	–	12	16	–	–
points	–	–	0	0	–	–	2	3	–	–
weighted points	–	–	0	0	–	–	0.28	0.48	–	–
Infection (n)	3	3	–	–	–	–	–	–	–	–

Table S1. *Cont.*

Study Name	Abou-assi et al. 2002 [23]		Eckerwall et al. 2007 [24]		McClave et al. 1997 [25]		Oláh et al. 2002 [26]		Petrov et al. 2013 [27]	
points	1	1	–	–	–	–	–	–	–	–
weighted points	0.09	0.09	–	–	–	–	–	–	–	–
Organ failure (n)	7	8	–	–	–	–	–	–	–	–
points	2	3	–	–	–	–	–	–	–	–
weighted points	0.18	0.27	–	–	–	–	–	–	–	–
Hosp. readmission (n)	–	–	2	3	–	–	–	–	1	2
points	–	–	2	3	–	–	–	–	1	4
weighted points	–	–	0.2	0.3	–	–	–	–	0.06	0.24
MOF or severity progression (n)	–	–	2	1	–	–	2	5	2	2
points	–	–	2	0	–	–	1	3	4	4
weighted points	–	–	0.2	0	–	–	0.14	0.48	0.24	0.24
VAS-pain	–	–	2	3	–	–	–	–	9	7
points	–	–	1	1	–	–	–	–	3	2
weighted points	–	–	0.1	0.1	–	–	–	–	0.18	0.12
Pain relapse (n)	1	–	1	4	–	–	–	–	1	8
points	0	–	0	1	–	–	–	–	0	4
weighted points	0	–	0	0.10	–	–	–	–	0	0.24
Nausea/vomiting (n)	–	–	13	21	–	–	–	–	0	6
points	–	–	2	3	–	–	–	–	0	1
weighted points	–	–	0.2	0.3	–	–	–	–	0	0.06
Antibiotics (n)	1	8	–	–	–	–	0	0	–	–
points	0	3	–	–	–	–	0	0	–	–
weighted points	0	0.27	–	–	–	–	0	0	–	–
Opiate-free treatment (n)	–	–	–	–	–	–	–	–	3	9
points	–	–	–	–	–	–	–	–	1	5
weighted points	–	–	–	–	–	–	–	–	0.06	0.3
Start of oral intake (day)	–	–	3	5	–	–	–	–	4	4
points	–	–	2	3	–	–	–	–	4	4
weighted points	–	–	0.2	0.3	–	–	–	–	0.24	0.24
Intervention (n)	2	–	6	7	–	–	5	11	8	9
points	0	–	2	2	–	–	1	2	4	5
weighted points	0	–	0.2	0.2	–	–	0.14	0.32	0.24	0.3
